# Regulation of cytochrome *c* oxidase activity by modulation of the catalytic site

**DOI:** 10.1038/s41598-018-29567-4

**Published:** 2018-07-30

**Authors:** Jacob Schäfer, Hannah Dawitz, Martin Ott, Pia Ädelroth, Peter Brzezinski

**Affiliations:** Department of Biochemistry and Biophysics, The Arrhenius Laboratories for Natural Sciences, Stockholm University, SE-106 91 Stockholm, Sweden

## Abstract

The respiratory supercomplex factor 1 (Rcf 1) in *Saccharomyces cerevisiae* binds to intact cytochrome *c* oxidase (Cyt*c*O) and has also been suggested to be an assembly factor of the enzyme. Here, we isolated Cyt*c*O from *rcf1*Δ mitochondria using affinity chromatography and investigated reduction, inter-heme electron transfer and ligand binding to heme *a*_3_. The data show that removal of Rcf1 yields two Cyt*c*O sub-populations. One of these sub-populations exhibits the same functional behavior as Cyt*c*O isolated from the wild-type strain, which indicates that intact Cyt*c*O is assembled also without Rcf1. In the other sub-population, which was shown previously to display decreased activity and accelerated ligand-binding kinetics, the midpoint potential of the catalytic site was lowered. The lower midpoint potential allowed us to selectively reduce one of the two sub-populations of the *rcf1*Δ Cyt*c*O, which made it possible to investigate the functional behavior of the two Cyt*c*O forms separately. We speculate that these functional alterations reflect a mechanism that regulates O_2_ binding and trapping in Cyt*c*O, thereby altering energy conservation by the enzyme.

## Introduction

The mitochondrial respiratory chain couples electron transfer to proton translocation across the inner membrane, thereby maintaining a proton electrochemical gradient that drives transmembrane transport as well as formation of ATP. The final electron acceptor is cytochrome *c* oxidase (Cyt*c*O), which catalyzes oxidation of the one-electron donor cytochrome *c* (cyt. *c*) and reduction of the four-electron acceptor O_2_ (for review, see e.g.^1,2^.). In *S. cerevisiae* the stability, assembly and activity of Cyt*c*O is regulated by at least three proteins, the respiratory supercomplex factors (Rcf) 1 and 2^3–6^, and the cytochrome oxidase interacting protein (Coi) 1^7^. The Rcf1 protein has multiple roles. It has been identified as an assembly factor of Cyt*c*O^8^, but it also acts to increase the relative concentration of the Cyt*c*O-cyt. *bc*_1_ supercomplex^3,4,6^. Additionally, Rcf1 regulates the activity of Cyt*c*O. Genetic removal of the protein results in a decrease in the O_2_-reduction activity to ~30% of that observed for Cyt*c*O in wild-type mitochondria^4,9^, presumably due to structural changes in a fraction of the Cyt*c*O population^10,11^. These structural changes are most noticeably reflected in changes in the kinetics of CO-ligand binding, which is accelerated by a factor of ~10^2^. Furthermore, data obtained with intact mitochondria suggested that a Cyt*c*O sub-population in the *rcf1*Δ mitochondria was reduced at lower redox potential than Cyt*c*O from the wild-type mitochondria^10^.

Upon purification of Cyt*c*O using affinity chromatography a mixture of two functionally distinct populations were isolated from the *rcf1*Δ mitochondria^11^. Here, we investigated ligand binding to heme *a*_3_ and internal electron transfer between hemes *a* and *a*_3_ as a function of the reduction pressure on the purified Cyt*c*O. The aim was to selectively reduce one of the two sub-populations of the *rcf1*Δ Cyt*c*O at a time such that the functional behavior of the two forms of the Cyt*c*O could be investigated separately.

Results from earlier studies have shown that incubation of Cyt*c*O under CO atmosphere, i.e. in the absence of O_2_, results in gradual reduction of the enzyme. In this redox reaction CO is oxidized to CO_2_ while two electrons are transferred to Cyt*c*O. Because at a pressure of 10^5^ Pa the CO concentration in solution is ~1 mM, a negligible amount of CO is consumed to reduce Cyt*c*O (µM concentration) in the above reaction. Upon reduction of heme *a*_3_ (and Cu_B_, which is reduced before heme *a*_3_), another CO molecule binds to the reduced heme *a*_3_ thereby increasing its apparent midpoint potential^12^. Consequently, the catalytic site becomes reduced (with CO bound to heme *a*_3_) while heme *a* and Cu_A_ remain oxidized, referred to as the “mixed-valence” state. The overall reaction is:

1 $${{\rm{Cu}}}_{{\rm{A}}}^{2+}{a}^{3+}{{\rm{Cu}}}_{{\rm{B}}}^{2+}{a}_{3}^{3+}\,+\,2\,{\rm{CO}}\,+\,{{\rm{H}}}_{{\rm{2}}}{\rm{O}}\,\to \,{{\rm{Cu}}}_{{\rm{A}}}^{2+}{a}^{3+}{{\rm{Cu}}}_{{\rm{B}}}^{+}{a}_{3}^{2+}-{\rm{CO}}\,+\,\,{{\rm{CO}}}_{2}\,+\,2{{\rm{H}}}^{+}$$

Because CO stabilizes the reduced state of heme *a*_3_, upon dissociation of the ligand an electron is transferred from heme *a*_3_ to heme *a*. With e.g. the *Rhodobacter sphaeroides* Cyt*c*O the time constant of this electron transfer is ~3 µs^13^, even though faster components have been identified^14^. The extent of the inter-heme electron transfer varies between Cyt*c*Os from different species depending on the relative midpoint potentials of hemes *a* and *a*_3_.

The data show that CO did reduce the catalytic site of the *S. cerevisiae* Cyt*c*O forming the mixed-valence-CO complex. In Cyt*c*O purified from the *rcf1*Δ mitochondria, initially CO reduced the catalytic site only in an enzyme sub-population that displayed the same behavior as Cyt*c*O isolated from the wild-type strain. Only upon further incubation we did observe reduction of a structurally altered sub-population. The difference in the midpoint potentials of the redox sites in the two Cyt*c*O sub-populations allowed us to investigate the functional behavior of the two Cyt*c*O forms separately. The data indicate that (*i*) the presence of Rcf1 stabilizes an intact form of Cyt*c*O, but Rcf1 is not strictly required for formation of this intact Cyt*c*O, and (*ii*) removal of Rcf1 results in a fraction Cyt*c*O with a lowered midpoint potential of the catalytic site, but an increased rate of ligand binding. These functional alterations suggest a mechanism to regulate O_2_ binding, reduction and energy conservation in the *S. cerevisiae* Cyt*c*O.

## Results

### Ligand binding

**Figure 1:**
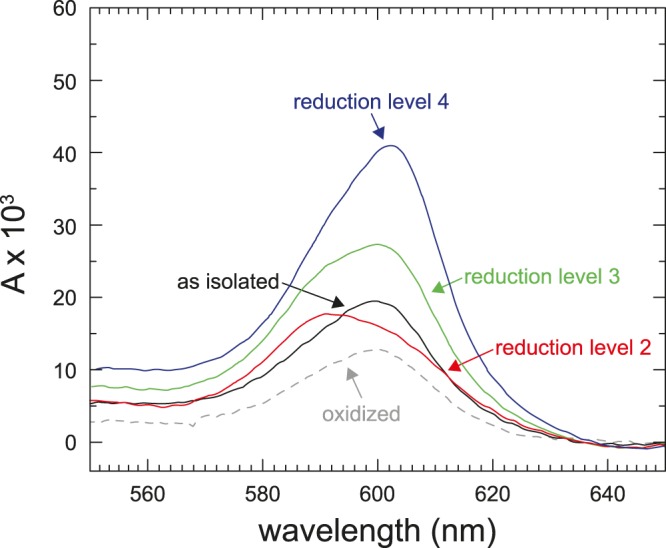
Absorption spectra of Cyt*c*O isolated from an *rcf1*Δ strain. The peaks at 602 nm and ~590 nm are associated with absorption by heme *a* (~80%) and the heme *a*_3_-CO complex, respectively. The spectrum of the “as isolated” Cyt*c*O (black) was taken under N_2_ atmosphere. The mixed-valence (2-electron reduced, reduction level 2) CO-bound form was obtained by incubation under CO atmosphere and careful titration with ferricyanide (red). The sample in reduction level 3 (~50% reduced heme *a*) was obtained after incubation with CO for 16 hours (green). The fully reduced Cyt*c*O (reduction level 4) was obtained after addition of 2 mM dithionite (dark blue). The spectrum of the oxidized Cyt*c*O was obtained upon addition of 1 mM ferricyanide to the “as-isolated” Cyt*c*O under air atmosphere (dashed line). Experimental conditions: ~1.5 µM Cyt*c*O, 150 mM KCl, 10% glycerol, 20 mM Hepes, pH 8.0 and 0.035% DDM, T ≅ 22 °C.

We prepared His-tagged (at Cox6) Cyt*c*O, isolated from the *rcf1*Δ strain, using affinity chromatography. Figure 1 shows absorbance spectra of the *rcf1*Δ Cyt*c*O before and after addition of CO and dithionite. The peak at 602 nm is associated mainly (80%) with absorption by reduced heme *a*. The fully oxidized form, obtained by addition of ferricyanide, is indicated by the dashed line in Fig. 1. This spectrum was determined only as a reference. Ferricyanide was not used in the experiments discussed below because it interferes with reduction by CO. Instead, we used the air-oxidized Cyt*c*O, “as isolated” Cyt*c*O, which contained ~20% reduced heme *a* (black spectrum in Fig. 1). Upon incubation with CO, the catalytic site (heme *a*_3_ and Cu_B_) was reduced and CO was bound to heme *a*_3_. Careful titration of this sample with ferricyanide yielded the mixed-valence-CO complex (red spectrum in Fig. 1). We refer to this state as “reduction level 2” to indicate that the Cyt*c*O fraction which is reduced carries on average two electrons per Cyt*c*O (see Discussion). Here, the 602-nm peak was smaller than in the other spectra because heme *a* was essentially fully oxidized (~90%). The peak at ~590 nm originates from the heme *a*_3_-CO complex.

When the mixed-valence state of Cyt*c*O was further reduced by long term incubation with CO (~16 hours), we observed reduction of heme *a* in ~50% of the population (green spectrum in Fig. 1). We refer to this state as “reduction level 3” to indicate that on average the Cyt*c*O is more reduced than in reduction level 2. Addition of dithionite resulted in full reduction of the Cyt*c*O with CO bound to heme *a*_3_ (dark blue spectrum), referred to as “reduction level 4”.

**Figure 2:**
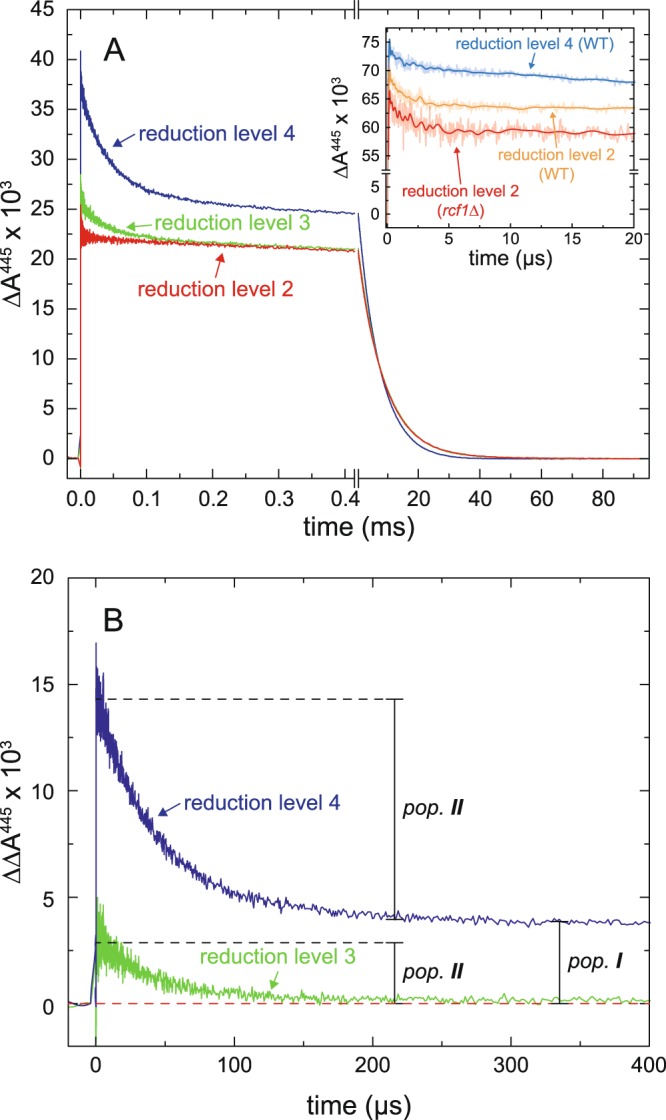
Absorbance changes at 445 nm associated with dissociation and recombination of CO from Cyt*c*O isolated from an *rcf1*Δ strain. (**A**) The reduction level of the samples is indicated in the graph. The same color code is used as for the spectra and conditions were the same as in Fig. 1. The inset shows absorbance changes monitored over a shorter time scale. The decay in absorbance with a time constant of ~2 µs seen with the mixed-valence Cyt*c*Os (orange and red traces for wild-type or *rcf1*Δ Cyt*c*O, respectively) is associated with electron transfer between hemes *a*_3_ and *a*. The larger slope observed with the fully reduced than with the mixed-valence Cyt*c*O is due to faster CO recombination in the former. (**B**) Additional absorbance changes upon forming reduction levels 3 and 4, respectively, from Cyt*c*O in reduction level 2. The absorbance changes are differences between the green and red, and blue and red changes in panel A, respectively. We interpret the two kinetic components in terms of two Cyt*c*O populations referred to as I and II, respectively. Experimental conditions were the same as in Fig. 1.

Figure 2A shows absorbance changes at 445 nm after light-induced CO dissociation of the mixed-valence Cyt*c*O (red trace). The initial increase in absorbance at *t* = 0 is associated with dissociation of the CO ligand, followed in time by a decrease in absorbance indicating recombination (CO ligand rebinding) with a time constant of 8.2 ± 0.3 ms (SD, *n* = 5), which was the only observed kinetic component over the shown time scale in the main panel. We refer to the population of Cyt*c*O that displays this 8.2-ms kinetic component as population I (indicated in Fig. 2B, see also the Discussion section). We observed also a small 2-µs component, seen in the inset and discussed below.

Next, we measured light-induced absorbance changes with the sample in which heme *a* was 50% reduced, i.e. at reduction level 3 (Fig. 1, green spectrum). As seen in Fig. 2A (green trace), pulsed illumination of this Cyt*c*O resulted in absorbance changes that were similar to those observed with the mixed-valence Cyt*c*O (reduction level 2). However, in addition to the 8.2-ms CO-recombination component, we observed a component with a time constant of 35 ± 2 µs (SD, *n* = 9) and an amplitude of 19 ± 6% (range of values, *n* = 2) of the total absorbance change. Figure 2B shows the difference between the green and red traces in panel A over a shorter time scale of 400 µs. This difference represents the absorbance changes, associated with ligand binding, after short-term and long-term incubation of the Cyt*c*O with CO. As seen in this figure, the further reduction of the sample resulted in appearance of only the rapid CO-recombination component with a time constant of ~35 µs. We refer to the Cyt*c*O subpopulation displaying the rapid component as population II (see Discussion).

Upon addition of dithionite all four redox sites of Cyt*c*O became reduced (blue spectrum in Fig. 1, reduction level 4). In this state the time constant of the slow component decreased slightly to 6.6 ± 0.5 ms (SD, *n* = 5) and the amplitude increased (Fig. 2, dark blue traces). The relative amplitude of the rapid component (τ = 39 ± 2 µs, SD, *n* = 5) increased to 33 ± 3% (SD, *n* = 3) of the total absorbance changes at 445 nm.

### Internal electron transfer

As described in the Introduction section, results from earlier studies have shown that after light-induced CO dissociation from the mixed-valence Cyt*c*O the electron at heme *a*_3_, stabilized by the binding of CO prior to the flash, equilibrates with heme *a* over a µs time scale. The inset of Fig. 2A shows absorbance changes at 445 nm over a shorter time scale of 20 µs. After the rapid increase in absorbance associated with CO dissociation we observed a decrease in absorbance with a time constant of ~2 µs, associated with electron transfer from heme *a*_3_ to heme *a* (see^13^) (orange trace). This rapid absorbance change was also observed with the fully reduced Cyt*c*O (light blue trace), but its amplitude was smaller. The observation of a small 2-µs component also with the fully-reduced Cyt*c*O presumably reflects transient CO binding to Cu_B_, which in the mixed-valence Cyt*c*O triggers the inter-heme electron transfer^14^. The 2-µs electron transfer was also observed with the mixed-valence Cyt*c*O isolated from the *rcf1*Δ mitochondria (Fig. 2A, inset over a shorter time scale than the main graph, red trace).

As seen in Fig. 2A, the CO recombination was slower (by ~20%, see above) with the mixed-valence than with the fully reduced Cyt*c*O. The reason is that in the former a fraction heme *a*_3_ is oxidized as a result of electron transfer to heme *a*, i.e. during the recombination process the fraction reduced heme *a*_3_ is decreased. The extent of heme *a*_3_ to heme *a* electron transfer was consistent with the difference in CO-recombination rates, which slows due to a fraction oxidized heme *a*_3_ during the rebinding process^13^.

### Effect of imidazole

**Figure 3:**
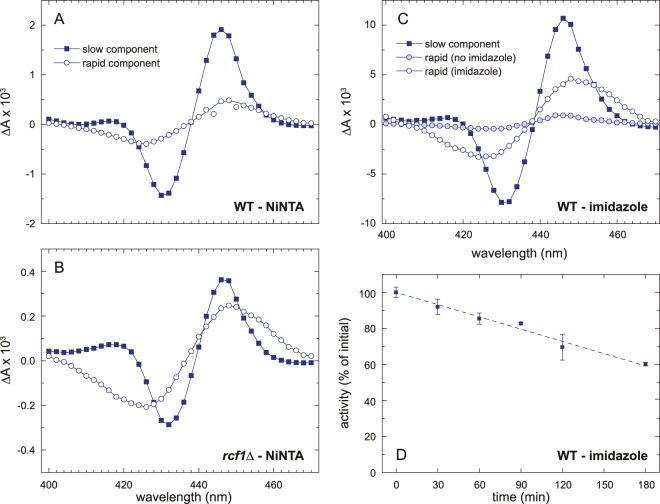
Ligand binding and activity of Cyt*c*O. The Cyt*c*O from the wild-type (**A**) and *rcf1*Δ (**B**) strains was purified using Ni-affinity chromatography. The protein was eluted with imidazole. Absorbance changes after flash-induced CO dissociation were measured in the wavelength range 400–470 nm. The traces were fitted with a sum of two exponentials and the amplitudes of the two components, rapid (τ ≅ 35 μs) and slow (τ ≅ 6 ms; open circles), are plotted. (**C**) Kinetic difference spectra for the slow and rapid components in the CO-recombination obtained at ~1 hour after after addition of 300 mM imidazole. Experimental conditions: ~0.1–0.5 µM Cyt*c*O, 4 mM sodium ascorbate, 2 μM PMS, 1.3 mM CO, 20 mM Tris, pH 7.5, 100 mM NaCl, 0.035% DDM, T ≅ 22 °C. (**D**) The O_2_-reduction activity of purified Cyt*c*O was measured at specific time points after addition of 300 mM imidazole. It was normalized to the amount of heme *a* in the sample at every time point (diminished with time as a result of precipitation). The initial rate just after addition of imidazole was set to 100%. Experimental conditions (panel D): 67 mM KP_i_, pH 6.8, 0.1 mM EDTA, 0.035% DDM, 20 mM ascorbate, 40 µM TMPD and 50 µM cyt. *c* from *S. cerevisiae*. The reaction was started upon addition of 10 nM Cyt*c*O (protein C-tagged).

In the present work we studied Cyt*c*O that was purified by means of Ni-affinity chromatography with a His_10_-tag on subunit Cox6. Imidazole was used for elution. This enzyme displayed a 35-µs CO-recombination component also when isolated from the wild-type mitochondria, suggesting structural changes. Furthermore, for Cyt*c*O isolated from the *rcf1*Δ strain the component was larger than that seen with the protein-C purified variant^11^. The kinetic difference spectra of the 35-µs and 6-ms components seen with the wild-type and *rcf1*Δ Cyt*c*O are shown in Fig. 3A,B. They are about the same as those observed for the *rcf1*Δ Cyt*c*O isolated using the protein-C tag. These results suggest that the exposure to imidazole induces the same structural alteration as removal of Rcf1 alone. To further investigate the effect of imidazole we incubated the wild-type Cyt*c*O isolated using a protein-C tag^11^ in a buffer containing imidazole (300 mM, at pH 8.9) for 1 hour and measured absorbance changes associated with CO recombination (Fig. 3C). As seen in the figure, the amplitude of the 35-µs component increased significantly. Next, we measured the O_2_-reduction activity of Cyt*c*O that had been incubated with imidazole. The CytcO purified from wild-type strains showed a decrease in activity, which after ~180 min yielded ~60% activity (Fig. 3D). Over the same time, we observed up to ~20% precipitation of the Cyt*c*O (the reduced-oxidized difference spectrum remained the same, but the amplitude of the peaks decreased over time, not shown). In the activity measurements we normalized the measured rates to the 602 nm absorbance for each time point.

We also tested the effect of addition of 300 mM imidazole (pH 8.9) on the activities of Cyt*c*Os (cyt. *aa*_3_) from *Rhodobacter sphaeroides* and from bovine heart (data not shown). In both cases we observed an initial drop in activity by ~15%, but the activity then remained unaltered, i.e. no time-dependent changes were observed.

The effect of imidazole was studied because both removal of Rcf1 and exposure of Cyt*c*O to imidazole yield similar effects, which allowed us to discuss the origin of the changes at a molecular level (see Discussion).

The Rcf1-Cyt*c*O interaction surface is not known, but data from a number of studies suggest that subunits Cox3, Cox12 and Cox13 are involved^3,4,6^. To exclude indirect effects of the removal of Rcf1 as a cause of the 35-µs component, we analyzed the Cyt*c*O for the presence of Cox12 and Cox13. Figure S1 shows a western blot confirming the presence of both subunits in the purified wild-type and *rcf1*Δ Cyt*c*Os.

### O_2_-reduction activity

**Figure 4:**
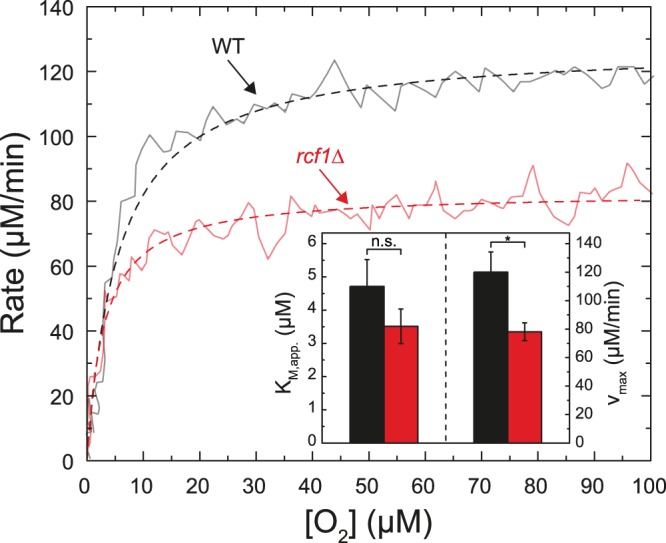
Oxygen-reduction rate. The O_2_-reduction rate as a function of time was determined using a Clark-type oxygen electrode. The starting O_2_ concentration was ~100 µM. For each O_2_ concentration the slope (rate) was determined. Data obtained with Cyt*c*O purified from the wild type (black) or *rcf1*Δ (red) strains are shown. The traces were fitted with a Michaelis-Menten equation (dashed lines). Parameters are shown in the inset. Experimental conditions were the same as in Fig. 3D, except that 1/2 of the buffer volume was incubated under an N_2_ atmosphere to yield lower starting O_2_ concentrations; *p < 0.025.

Results from earlier studies have shown that O_2_ binds weakly and reversibly to the catalytic site of Cyt*c*O, but is trapped by rapid electron transfer from heme *a* to heme *a*_3_ yielding an apparent high affinity for O_2_^15^. The K_M_, i.e. the O_2_ concentration at which the turnover rate is 1/2 of the maximum rate, was found to be in the µM range^16^. Here, we determined the K_M_ values for Cyt*c*O isolated from the wild-type and *rcf1*Δ mitochondria. Figure 4 shows the O_2_-reduction rate as a function of O_2_ concentration for [O_2_] ≤ 100 µM (slope of [O_2_] as a function of time). Both traces could be fitted with a Michaelis-Menten equation (dashed lines in Fig. 4) where the K_M_ values were 4.7 ± 0.8 µM and 3.5 ± 0.6 µM for the wild-type and *rcf1*Δ Cyt*c*Os, respectively (SD, *n* = 3). The maximum rates were 120 ± 14 nmol O_2_ ml^−1^min^−1^ (800 s^−1^) and 78.2 ± 6.3 ml^−1^min^−1^ (520 s^−1^), respectively (SD, *n* = 3). The decrease in the maximum turnover rate upon removal of Rcf1 in the detergent-purified Cyt*c*O is consistent with earlier observations^11^.

## Discussion

We have investigated functional characteristics of the two Cyt*c*O sub-states isolated using affinity chromatography from the *rcf1*Δ mitochondria. As already outlined above, results from earlier studies have shown that a typical feature of structural changes in Cyt*c*O, caused by the removal of Rcf1, is a rapid CO-recombination component with a time constant of 35 µs. This kinetic component is ~10^2^ times faster than that of CO binding to intact Cyt*c*O (τ ≅ 6 ms). In mitochondria isolated from the *rcf1*Δ strain the rapid component comprised ~60% of the total absorbance change at 445 nm^9^. Because a fraction of the structurally altered Cyt*c*O did not bind to the affinity column, the relative amplitude of this component in the purified Cyt*c*O was smaller (~30% at 445 nm)^11^.

As seen in Fig. 2A, the rapid component was not observed under conditions when only the mixed-valence Cyt*c*O was formed (red trace), i.e. in a state in which the catalytic site is reduced while heme *a* and Cu_A_ are oxidized. The rapid CO-recombination component appeared only upon further reduction or addition of dithionite (see Fig. 2B). As discussed above, upon incubation of the oxidized Cyt*c*O with CO, each Cyt*c*O accepts two electrons from a CO molecule, while another CO molecule binds to heme *a*_3_ to form the mixed-valence-CO complex. At neutral pH, this complex is typically formed within one hour of incubation at room temperature (see^12^ for data with the bovine heart Cyt*c*O). Further incubation results in gradual reduction of heme *a*, but the fully (4-electron) reduced state is never achieved.

The data show that prolonged incubation of the Cyt*c*O resulted in a ~50% reduction of heme *a* (Fig. 1, green spectrum). The amplitude fraction of the rapid CO-recombination component was ~19% (Fig. 2, green trace). Upon full reduction of Cyt*c*O by addition of dithionite (Fig. 1, blue spectrum) the amplitude fraction of the rapid component increased to ~33% (Fig. 2, dark blue trace). Thus, these results indicate that the amplitude of the rapid component approximately (within the error, see Results) scales with the reduction level of heme *a*.

We offer two explanations for the observed behavior: (*i*) the 35-µs component is consequence of reduction of heme *a*, i.e. it is not seen with the mixed-valence Cyt*c*O because in this state heme *a* is oxidized. This explanation is consistent with the correlation of the relative contribution of the 35-µs component to the total absorbance change and the degree of heme *a* reduction as outlined above. (*ii*) The rapid component originates from a fraction Cyt*c*O in which the midpoint potential of the catalytic site is lowered (population II) or in which reduction by CO is kinetically impaired. In other words, initially only the intact fraction Cyt*c*O would be reduced by CO (population I) and after prolonged incubation (and addition of dithionite) also an increasing fraction of the structurally altered Cyt*c*O would be reduced along with reduction of heme *a* in both Cyt*c*O populations.

**Figure 5:**
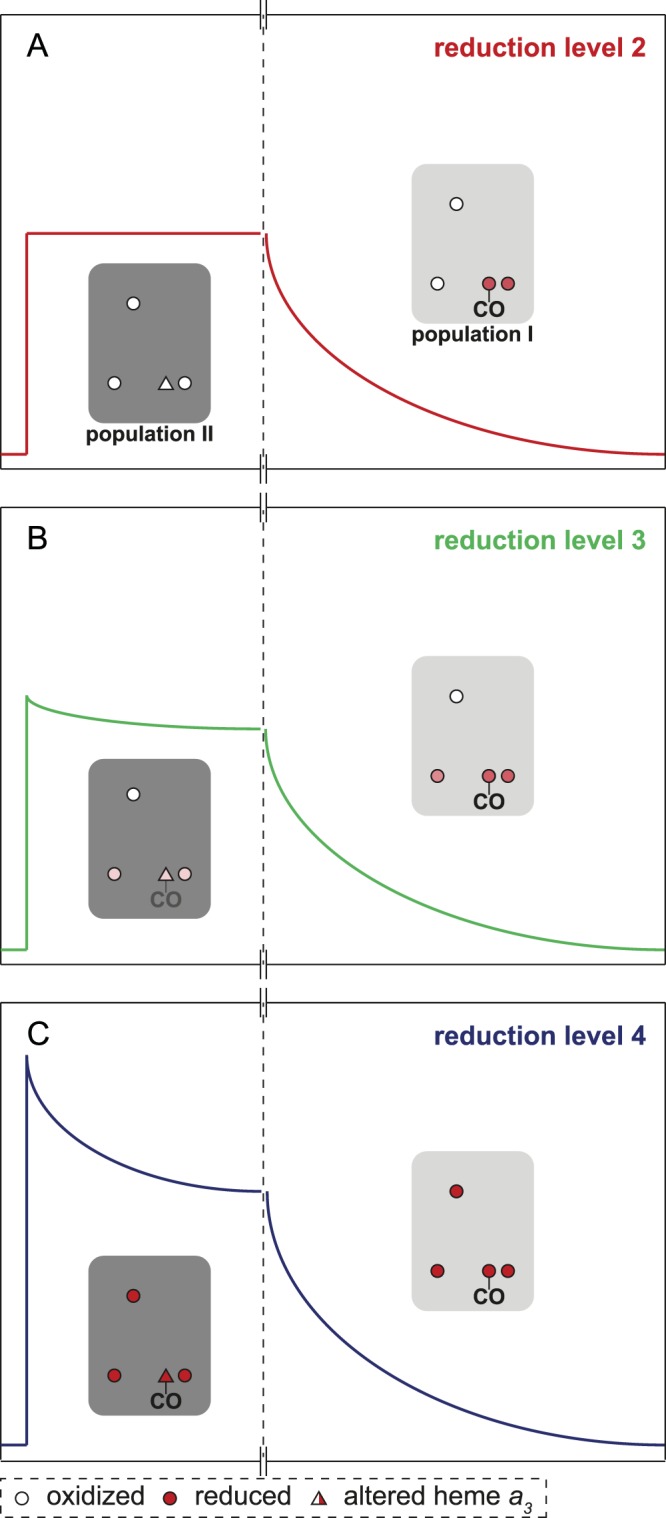
Schematic model. Two Cyt*c*O populations are suggested, I and II, based on the data from the flash photolysis experiment. (**A**) Reduction level 2, i.e. the 2-electron reduced Cyt*c*O of population I (red). Only population I is reduced and it displays the same CO-recombination time constant as the wild-type Cyt*c*O, i.e. it forms the mixed-valence state with CO bound to heme *a*_3_. Light-induced dissociation of this state results in the absorbance changes schematically shown on the long time scale (right). Under conditions when the mixed-valence CO-bound state is formed in population I, the other population II, which is structurally altered, remains oxidized and does not contribute to the observed signals. (**B**) Reduction level 3 (green). After further incubation under reductive conditions about 50% of heme *a* becomes reduced. Now also population II becomes partly reduced. Here, a rapid component starts appearing (short time scale, left), which is associated with CO recombination with population II. (**C**) Reduction level 4, i.e. the fully reduction Cyt*c*O (blue). All redox centers in both populations are reduced. Populations I and II display the slow and rapid CO-recombination components, respectively. Empty and filled circles represent oxidized and reduced, respectively redox sites. Stronger red color indicates a larger fraction reduced site.

We note that explanation (*i*) would imply that reduction of heme *a* results in structural changes at the catalytic site, which we find unlikely. Furthermore, results from earlier studies showed that a fraction of Cyt*c*O in mitochondria isolated from the *rcf1*Δ strain could not be reduced by ascorbate^10^, which suggests that in these mitochondria a fraction of the Cyt*c*O hemes are in a structurally altered environment and thus exhibit a lower midpoint potential. These observations support explanation (*ii*) above. This scenario is also summarized schematically in Fig. 5 and described in detail in the figure legend. In short, we suggest that initially only the intact Cyt*c*O is reduced (i.e. population I) by two electrons to form the mixed-valence state in which heme *a*_3_ and Cu_B_ are reduced (with CO bound to heme *a*_3_) while heme *a* and Cu_A_ are oxidized. This enzyme population displays the slow CO-recombination component. Above, we referred to this state as “reduction level 2” to indicate that the Cyt*c*O in population I forms the 2-electron reduced mixed-valence state, but population II remains oxidized. After further incubation under reductive conditions the structurally altered population (II) becomes reduced (reduction level 3) and binds CO, which results in appearance of the rapid CO-recombination component. Here, the average reduction level of heme *a* is 50%, but most likely heme *a* in populations I and II is reduced to different degrees. The most important difference between the samples in reduction levels 2 and 3 is that only in the latter case the catalytic site of population II becomes reduced, which leads to appearance of the rapid CO-recombination component. Upon addition of dithionite both populations I and II become fully reduced to form the Cyt*c*O-CO-complex. Under these conditions populations I and II yield the slow and rapid CO-recombination components, respectively.

Next, we address the accelerated CO binding in the structurally altered Cyt*c*O (population II). Binding of CO from solution to heme *a*_3_ involves transient binding to Cu_B_; the observed CO-recombination rate is determined by both the fraction bound CO to Cu_B_ and the rate by which CO is repositioned from Cu_B_ to heme *a*_3_^17^. Thus, as discussed previously^9^, accelerated CO recombination in a fraction of Cyt*c*O isolated from the *rcf1*Δ strain is indicative of structural changes around the Cu_B_ site. These structural changes would either act to accelerate CO repositioning from Cu_B_ to heme *a*_3_, to increase the fraction CO-bound Cu_B_ or promote direct CO recombination from solution to heme *a*_3_.

Any structural changes around the Cu_B_ site would also result in changes around heme *a*_3_ as the two sites are located in close proximity^18^. A decreased heme *a*_3_ midpoint potential suggests a more polar environment or an increased water access to the catalytic site^19^. This observation is also consistent with the blue-shift in the reduced minus oxidized and CO-reduced minus reduced difference spectra of heme *a*_3_^11^ because an increase in the solvent polarity typically results in in a blue-shift of the absorption maxima as evidenced from studies on myoglobin and hemoglobin^20^.

A rapid 35-µs component in the CO-recombination reaction was also observed with wild-type Cyt*c*O that was incubated with imidazole (Fig. 3) and in Cyt*c*O isolated from *rcf1*Δ mitochondria the amplitude of the rapid component was larger after incubation with imidazole. Furthermore, we observed a gradual loss in activity after incubation with imidazole. Imidazole has been shown to inhibit cyt. *bc*_1_ by binding to the heme of cyt. *c*_1_, which also resulted in a lower midpoint potential of the heme^21^. In the present study, the effect of addition of imidazole was very similar to that of Rcf1 removal, which further supports that in a sub-population of the Cyt*c*O isolated from *rcf1*Δ mitochondria the structure of the heme *a*_3_ protein environment is altered.

A question arises whether or not changes in the midpoint potential and ligand-binding kinetics would have any functional significance. As already outlined above, results from earlier studies showed that an apparent small *K*_M_ for O_2_ binding is result of a weak (cf. large *K*_M_) and reversible binding of O_2_ to the catalytic site, and trapping of O_2_ by rapid electron transfer from heme *a* to heme *a*_3_^15^. It was suggested that this mechanism is a strategy aimed at avoiding investment free energy in tight O_2_ binding, but rather trap O_2_ kinetically at the catalytic site^16^. As seen in Fig. 4, we did not observe any significant difference in the K_M_ values. Assuming that an increased CO-binding rate in a sub-population of *rcf1*Δ Cyt*c*O also implies an accelerated O_2_ binding, we speculate that in this sub-population O_2_-binding is tighter (assuming that O_2_ dissociation from heme *a*_3_ remains unaltered). At the same time the lower midpoint potential of the catalytic site would limit the degree of kinetic trapping of the bound O_2_ (assuming slower electron transfer upon decreasing the driving force). In other words, in a fraction of the *rcf1*Δ Cyt*c*O the O_2_-reduction mechanism would yield a lower degree of energy conservation, which may be advantageous if external conditions are such that the organism would benefit from an increased heat production or a larger degree of fermentation.

At present we cannot explain the effect of removing Rcf1 at a molecular level. The data in Figure S1 show that subunits Cox12 and Cox13 remain bound to the Cyt*c*O from *rcf1*Δ mitochondria, i.e. the functional behavior of Cyt*c*O from these mitochondria is not a consequence of losing Cox12 or Cox13. We note that the recently-determined structure of Rcf1^22^ shows a dimer with an unusually charged dimer interface. Most likely the monomeric form of Rcf1 interacts with Cyt*c*O^22^, possibly via the charged domain. As already noted above, results from earlier studies indicate that Rcf1 interacts with subunits Cox12 and Cox13^3,4,6^, located near Cox3 that forms a cleft leading through the membrane-spanning part of Cyt*c*O to the catalytic site. In other words, binding of Rcf1 may occur in sufficient vicinity to the catalytic site to modulate its structure. Hopefully, future structural studies of the Cyt*c*O-Rcf1 complex will offer insights into possible functional effects of this interaction.

In conclusion, the data indicate that removal of Rcf1 results in two Cyt*c*O sub-populations that could be purified in a mixture and then kinetically dissected and studied. One of these sub-population displayed unperturbed absorption spectra, CO-recombination kinetics and internal electron transfer (Fig. 2 and^11^). In the other population the midpoint potential of the catalytic site was lowered and CO recombination was accelerated. Thus, the data reveal structural changes in Cyt*c*O that result from removal of Rcf1. On the other hand, the data also show that Rcf1 is not strictly required for correct assembly/function of the Cyt*c*O, but removal of Rcf1 yields a fraction of Cyt*c*O that has a structurally altered heme *a*_3_ protein environment and consequently, an altered functional behavior.

## Materials and Methods

### Cell growth

The Cyt*c*O was purified from two different *S. cerevisiae* strains: W303a Cox6_His10_ *rcf1Δ* as well as that described in^11^. The gene *rcf1* was deleted by homologous recombination using a *kanMX4* cassette. To construct the His10-tagged variant of Cox6, the stop codon of the endogenous *ORF* was replaced by a His10-tag followed by a *trp1* selection cassette. A volume of 5 ml YP medium (1% yeast extract, 2% peptone), supplemented with 2% galactose were inoculated and grown at 30 °C and 160 rpm. After 24 h, cells were diluted to 100 ml medium and grown for another 16 h under the same conditions. Cells were then transferred into 200 ml medium and incubated for 8 h at 30 °C while shaking at 160 rpm. Finally, cells were transferred to 2 l medium. After 16 h of incubation, the cells were harvested by centrifugation at 6500 × g (5 min, 4 °C). The cells were then re-suspended in 50 mM KP_i_, pH 7.0 and pelleted at 6500 × g for 5 min at 4 °C before cell disruption.

### Preparation of mitochondrial membranes

Mitochondrial membranes were prepared as in^11^. In short, cells were disrupted in a Constant Cell Disrupter (Constant Systems). Cell debris was then removed by centrifugation, before membranes were pelleted by ultra-centrifugation and washed in several peletting/resuspension steps. The membranes, re-suspended in a final buffer, were shock-frozen in liquid N_2_ and stored at −80 °C.

### Purification of CytcO

The Cyt*c*O was purified using two different protocols as described briefly below.

For experiments in which reduction of heme *a*_3_ and CO ligand binding were studied, Cyt*c*O was purified using a protocol modified from^23^. Briefly, mitochondrial membranes were diluted to a concentration of 10 mg protein/ml in 50 mM KP_i_, pH 8.0. The the sample was incubated with 2% (w/v) DDM (*n*-dodecyl *β*-D-maltoside; Glycon) for 1 h on ice. Remaining membrane fragments were removed by centrifugation (15 000 × g,10 min, 4 °C). The cleared lysate was incubated with Ni^2+^-nitrilotriacetic acid (Ni-NTA) agarose for 2 h at 4 °C while tumbling. The protein-bound resin was washed with 10 column volumes of 150 mM KCl, 10% glycerol, 20 mM Hepes, pH 8.0, 10 mM imidazole and 0.035% (w/v) DDM. Cyt*c*O was eluted by incubation with one column volume 150 mM KCl, 10% glycerol, 20 mM Hepes, pH 8.0, 300 mM imidazole and 0.035% (w/v) DDM for 30 min. The elution step was repeated two more times. Imidazole in the buffer was removed by centrifugation (Amicon Ultra Centrifugal Filter Unit, 50 kDa cut-off) and dilution in 150 mM KCl, 10% glycerol, 20 mM Hepes, pH 8.0 and 0.035% DDM. The final Cyt*c*O concentration was adjusted to 1–2 µM Cyt*c*O. Samples were frozen in liquid N_2_ and stored at −80 °C until use.

For experiments in which the effect of imidazole was investigated and O_2_-reduction rate was measured, Cyt*c*O was purified from wild type or *rcf1*Δ strains by Protein C affinity chromatography as reported in^11^. Also the western blot analysis was performed with this Cyt*c*O. Briefly, mitochondrial membranes were incubated with 2% (w/v) DDM and cell debris was removed. The cleared lysate was incubated on a Protein C matrix for 1 hour at 4 °C. Cyt*c*O was eluted by several incubation steps with buffer containing 5 mM EDTA. During incubation with imidazole the pH was 8.9.

### Flash Photolysis

The purified enzyme was transferred to a Thunberg cuvette in which the atmosphere was exchanged to N_2_, followed in time by exchange of the atmosphere for CO. The sample was incubated overnight at 4 °C until heme *a*_3_ and Cu_B_ were reduced (mixed-valence state). Continued incubation in CO resulted in fractional reduction of heme *a*. If necessary, this state could be converted back to the mixed-valence state by addition of ~100 nM sodium ferricyanide. For observations of the fully reduced state sodium dithionite was added (a few µl from a concentrated, buffered solution) to reach a concentration of 2 mM. The different oxidation states were analyzed spectrophotometrically.

The kinetics of CO recombination to Cyt*c*O, after laser-flash induced dissociation of the ligand (~10 ns laser flash, λ = 532 nm, Nd-YAG laser, Quantel), was measured at different wavelengths as described previously^11^. The changes in absorbance were recorded on a flash-photolysis setup (Applied Photophysics, UK). Results were analyzed using ProK (Applied Photophysics, UK) and Origin 2016 software (OriginLab, USA).

### Oxygen reduction

Oxygen consumption of purified Cyt*c*O was determined using a Clark-type oxygen electrode. First, a baseline was recorded with buffer containing 67 mM KP_i_, pH 6.8, 0.1 mM EDTA and 0.035% DDM. Then, we added 20 mM ascorbate, 40 µM *N,N,N′,N′-tetramethyl-p-phenylenediamine* (TMPD) and 50 µM cyt. *c* from *S. cerevisiae*. The reaction was started by addition of 10 nM Cyt*c*O. To measure O_2_ reduction with a low initial O_2_ concentration, 1/2 of the buffer volume was incubated under N_2_ atmosphere prior to the experiment and then mixed with the air-equilibrated remaining part of the buffer solution, yielding ~100 µM O_2_.

### Western Blot analysis

Proteins were separated by 16% acrylamide/0.2% bisacrylamide SDS PAGE and transferred to a nitrocellulose membrane (Carl Roth, Germany).

## Electronic supplementary material

Supplementary information
