# Supplementary material for: Regulation of cytochrome c oxidase activity by modulation of the catalytic site
Source: Sci Rep. 2018 Jul 30;8:11397. doi: 10.1038/s41598-018-29567-4 (PMC6065377; doi:10.1038/s41598-018-29567-4)
Supplement: Supplementary file 1 — Supplementary information [file 41598_2018_29567_MOESM1_ESM.docx]

Supplementary Information

Regulation of cytochrome *c* oxidase activity by modulation of the catalytic site

Jacob Schäfer, Hannah Dawitz, Martin Ott, Pia Ädelroth and Peter Brzezinski*

Department of Biochemistry and Biophysics, The Arrhenius Laboratories for Natural Sciences, Stockholm University, SE-106 91 Stockholm, Sweden.

Key words: electron transfer, cytochrome *aa*_3_, membrane protein, ligand, kinetics, mechanism, imidazole.

^*^ Correspondence: peterb@dbb.su.se, fax: +46-8-153679, phone +46 70 609 2642

#
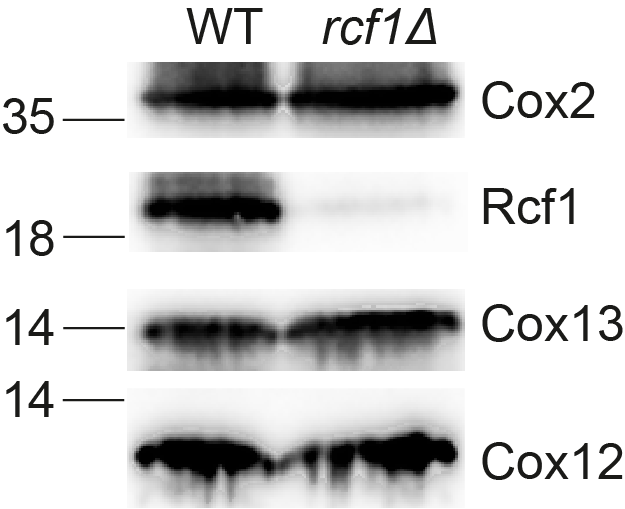


**Figure S1A** Western blot analysis of Rcf1 as well as Cyt*c*O subunits Cox2, Cox12 and Cox13, done with Cyt*c*O purified from the wild-type and *rcf1*Δ strains. Experimental conditions: 16% acrylamide/2% bisacrylamide SDS PAGE, blotted on a nitrocellulose membrane. For preparation of the Cyt*c*Os, see the “Materials and Methods” section of the main paper.


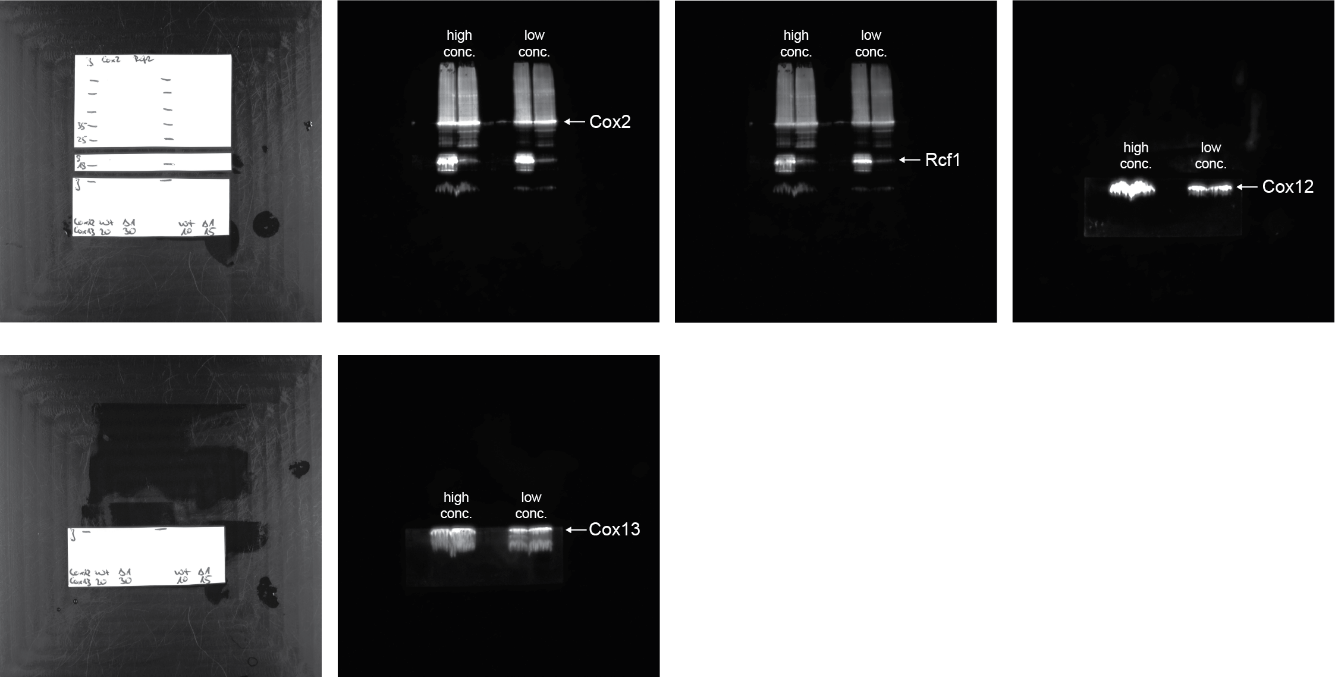


**Figure S1B** **Western Blot original pictures**. Samples were loaded in two different concentrations (high and low, as indicated in the figure). The bands shown in panel A are indicated with arrows (only low concentration was used).
